# Evidence gaps and route-dependent development requirements for CAR cellular therapy in urothelial carcinoma: a systematic evidence map and public-data analysis

**DOI:** 10.3389/fimmu.2026.1941701

**Published:** 2026-10-22

**Authors:** Jiayi Liu, Zhibiao Guo, Jinke Guo, Shaoqiang Xing, Qian Liu

**Affiliations:** 1 The First Central Clinical College of Tianjin Medical University, Tianjin, China; 2 Department of Urology, Tianjin First Central Hospital, Tianjin, China

**Keywords:** bladder cancer, CAR T cell, CAR-NK cell, chimeric antigen receptor, evidence map, intravesical delivery, single-nucleus RNA sequencing, urothelial carcinoma

## Abstract

**Background:**

Chimeric antigen receptor (CAR)-engineered cells are being investigated for urothelial carcinoma, but evidence requirements for antigen selection and local or systemic delivery remain undefined. We asked whether any target or route can presently be prioritised.

**Methods:**

We combined a systematic evidence map and registry audit with secondary analysis of public bulk and single-nucleus transcriptomes and normal-tissue resources. In GSE169379, infercnvpy used same-object non-epithelial reference cells; epithelial nuclei with high copy-number variation (CNV-high) scores were defined as those above the 99th percentile of reference scores. Aggregate rankings were replaced by two evidence gates: antigen eligibility and then product- and route-specific exposure validation. No new participants or experiments were included.

**Results:**

All 13 peer-reviewed primary studies were preclinical; none reported a treated patient with urothelial carcinoma. Five registry programmes were retained without bladder-specific results; the only urothelial-specific record changed from recruiting to terminated. Among 21,756 sampled epithelial nuclei from the 29,974-nucleus GSE169379 tumour object, 1,828 were classified CNV-high using 8,218 non-epithelial nuclei from the same object as the reference. Across 18 evaluable patients, median detection fractions were 0.083 for NECTIN4, 0.169 for ERBB2, 0.229 for CD44, 0.267 for EGFR, 0.385 for PSCA and 0.093 for MUC16; corresponding medians in four normal-urothelial batches were 0.290, 0.401, 0.423, 0.598, 0.527 and 0.008. These unpaired cross-object RNA contrasts do not establish a therapeutic window. Equal-dose intravesical MUC16-directed CAR T cells outperformed intravenous administration in two orthotopic xenograft models and showed bladder-localised exposure, but this single programme cannot establish a general route advantage or human safety.

**Conclusions:**

No evaluated target meets requirements for unqualified clinical prioritisation. Epitope-matched malignant-cell surface expression and functional normal-tissue safety should precede testing whether a specific route achieves the exposure required by the intended disease distribution. Current route evidence is product-specific and preclinical.

## Introduction

Chimeric antigen receptor (CAR)-engineered immune-cell therapies have changed the treatment of several haematological malignancies, yet their translation to solid tumours remains constrained by antigen heterogeneity, limited trafficking, functional suppression and on-target/off-tumour toxicity. These barriers are commonly discussed as properties of solid tumours in general. However, the anatomical setting in which a CAR product must operate is not uniform across cancers—or across stages of the same cancer. Urothelial carcinoma provides an unusually informative test case because organ-confined bladder tumours are accessible through the urinary lumen, whereas locally advanced and metastatic disease requires control across multiple systemic sites (1).

Antigen engagement on malignant cells, surface density above the activation threshold, normal-tissue specificity, escape and effector fitness are prerequisites for CAR activity. Delivery route does not change whether a CAR recognises its target. It can, however, alter the concentration, residence and systemic dissemination of an otherwise eligible product. These are sequential questions: an antigen must first satisfy a biological eligibility gate, after which the route must be tested against the intended distribution of disease and acceptable exposure pattern.

The route question has been tested directly in only one peer-reviewed bladder programme. Intravesical MUC16-directed, mesothelin-based CAR T cells were compared with the same intravenous dose in orthotopic bladder-cancer models (2). Separate conference reports described intravesical NECTIN4-directed CAR T cells and bladder-directed CAR-NK constructs but did not provide a peer-reviewed route comparison (3–5). Trial registries contain one urothelial-carcinoma-specific CAR T-cell programme and several pan-solid-tumour programmes permitting bladder-cancer enrolment (6–10), but none has posted a bladder-specific outcome. A nominally recruiting urothelial-carcinoma CAR-NK record was excluded because its official summary still described the study as hypothetical and as an example (11).

We therefore did not treat anatomical compartment as a surrogate for CAR efficacy or as a replacement for antigen qualification. We mapped the CAR evidence in urothelial carcinoma, quantified candidate-antigen expression in bulk and single-nucleus datasets, added copy-number variation (CNV)-based malignant-cell-compatible inference, audited normal-tissue liabilities and updated clinical registries. Our objective was to determine whether any target or route could presently be prioritised and, if not, to define an auditable sequence of evidence requirements for antigen selection and route-specific development.

## Methods

### Study design and protocol

We designed a systematic evidence map with reproducible secondary analysis of public molecular data. The protocol, eligibility criteria and candidate-antigen panel were prespecified on 15 July 2026 before quantitative results were inspected; deviations were recorded. The study was not an intervention-effect review, and no pooled treatment-effect meta-analysis was planned. During revision, we removed aggregate ordinal target and platform-route scores because the available non-comparative inputs did not justify a quantitative hierarchy.

### Literature search

MEDLINE/PubMed was searched from database inception using controlled vocabulary and title/abstract terms for bladder neoplasms or urothelial carcinoma combined with chimeric antigen receptors, CAR T cells, CAR-NK cells and related engineered immune-cell platforms. Supplementary searches addressed intravesical or locoregional administration, candidate antigens and review overlap. Crossref and OpenAlex were used for supplementary discovery and digital object identifier (DOI) verification. The searches were locked on 17 July 2026 and rerun through the official PubMed, Crossref, OpenAlex and ClinicalTrials.gov application programming interfaces on 27 August 2026. Reference lists and forward citations of included primary studies and recent reviews were screened. Complete executable queries, dates, counts, dated raw responses and deduplication rules are provided in **Supplementary Methods 1** and the public repository.

### Eligibility and study selection

We included peer-reviewed *in-vitro*, organoid, *in-vivo*, translational and clinical studies that evaluated a CAR-engineered immune-cell platform in bladder cancer or urothelial carcinoma. Registered interventional trials were included irrespective of recruitment status. Conference abstracts were retained only when no corresponding full report was identified and were labelled separately throughout. Reviews were used for citation chaining and overlap analysis but did not substitute for primary evidence. Studies of conventional NK cells, tumour-infiltrating lymphocytes, T-cell receptor-engineered cells or immune engagers without a CAR component were excluded except when used as contextual comparators. Upper-tract urothelial-carcinoma evidence was recorded separately and was not combined with bladder-specific quantitative analyses unless separable data were available.

Records were deduplicated by DOI, followed by PubMed identifier and normalised title. Jiayi Liu and Zhibiao Guo independently screened the records at title/abstract and full-text stages against the prespecified eligibility criteria. Screening decisions were reconciled by discussion; exclusion reasons were recorded at full-text review. During reconciliation and supplementary discovery, the reviewers identified one eligible Nectin4 CAR-T Original Article outside the locked database exports. Its publisher full text was verified, and the report was added to the evidence base. Companion abstracts, trial records and full publications were linked to prevent double counting.

### Evidence extraction and maturity assessment

For each included record, we extracted disease state, antigen, effector-cell platform, donor source, CAR architecture, administration route, model system, comparator, sample size, safety observations, antitumour readouts, publication status and availability of human outcomes. Evidence was grouped descriptively as cell line, three-dimensional culture or organoid, subcutaneous xenograft, orthotopic or syngeneic model, registry record, or peer-reviewed human outcome. These non-comparative categories were not aggregated into a numerical maturity score.

### Clinical-trial registry audit

ClinicalTrials.gov was searched using disease, platform and candidate-antigen terms and updated on 27 August 2026. We extracted registry identifier, official title, eligibility, disease compartment, antigen, cell source, CAR architecture, administration route, lymphodepletion, dose and schedule, phase, recruitment status, planned enrolment, dates, locations, outcome measures and availability of posted or peer-reviewed results. Historical changes in recruitment status or study dates were inspected for trials with long intervals between first registration and reported study initiation. Registry entries were treated as programme metadata and not as evidence that dosing, activity or efficacy occurred.

### Candidate-antigen panel

The prespecified gene-level panel comprised NECTIN4, ERBB2, MUC16, MUC1, CD44, EGFR, PSCA, MSLN, CD276, MET, FOLR1, FOLR2 and ROR2. Non-gene-defined targets, including tumour-associated glycoforms, were assessed from pathology and functional evidence rather than inferred from bulk gene expression. Because CD44v6 is an alternatively spliced isoform, gene-level CD44 abundance was not treated as a quantitative surrogate for CD44v6 surface expression.

### Bulk tumour and normal-tissue expression

Muscle-invasive bladder-cancer expression data were obtained from the UCSC Xena TCGA-BLCA HiSeqV2 matrix (12). After barcode-based sample classification, 407 primary tumours and 19 adjacent-normal bladder samples were retained. Metastatic urothelial carcinoma was assessed using the GSE176307 Salmon TPM matrix (13). Of 92 RNA-sequencing libraries, 90 mapped to 89 patients through the deposited sample key; two libraries without a key were excluded, and two libraries from one patient were averaged on the TPM scale before log2 transformation. No eligible, reproducibly processed non-muscle-invasive cohort entered the primary analysis.

Normal-tissue evidence was assessed using current Human Protein Atlas tissue-level and cell-type-level RNA and protein records (14). A harmonised TCGA/GTEx sensitivity analysis was prespecified but was not performed in this study. Expression in normal urothelium was reported separately from safety-critical systemic tissues. Data from different assay platforms were not naively merged, and cross-cohort conclusions required directional consistency rather than a pooled expression value.

Normal-tissue liability was adjudicated using a prespecified hierarchy that separated RNA abundance, immunohistochemical protein localisation, antibody reliability, target isoform or glycoform, epitope accessibility and reported functional toxicity. A favourable tumour-to-normal RNA ratio was not considered sufficient evidence of safety when surface protein was present in a vital normal tissue. Local administration was treated as an exposure-mitigation hypothesis rather than evidence that off-tumour risk was absent.

Within each cohort, we reported continuous expression, the proportion of samples above prespecified sensitivity thresholds, interquartile range and median absolute deviation. Antigen co-expression was assessed at sample level. Because the TCGA and GSE176307 matrices used different expression scales, each cohort was analysed separately with scale-specific threshold grids; absolute threshold-defined prevalence was not pooled across cohorts. Threshold-dependent antigen-pair results were treated as sensitivity analyses rather than primary evidence. Older matrices used PVRL4 for NECTIN4, which was mapped prospectively during preprocessing and retained in the provenance record.

### Single-nucleus expression and CNV-high epithelial inference

The prespecified single-cell candidates were GSE135337, GSE169379 and GSE222315. Inclusion in the cell-type synthesis required verifiable deposited cell-level annotations. GSE135337 comprised seven tumour matrices and one paracancerous matrix but had no deposited cell-type labels; we therefore restricted it to sample-level raw-count detection-sensitivity summaries (15). GSE169379 comprised a treatment-naive muscle-invasive bladder-cancer H5AD with 67,988 nuclei, 25 patient identifiers and source cell-type labels, and a histologically normal bladder H5AD with 25,102 nuclei and four deposited batch identifiers. The GEO study design reports that the normal object was derived from three patients (16). Because copy-number inference was completed in GSE169379, GSE222315 was not included in downstream analyses.

Deposited filtered matrices and source annotations were used without doublet removal, reclustering or *de novo* cell-type assignment. For the descriptive GSE169379 analyses, raw counts were library-size normalised per nucleus and transformed as log(1+counts per 10,000). Source labels were pooled into epithelial/urothelial, myeloid/mast, lymphoid, endothelial, stromal and other compartments. Counts were aggregated within each deposited patient for the tumour cohort and within each deposited batch for the normal cohort; medians were then calculated across source units.

For copy-number inference, we sampled up to 1,000 epithelial nuclei per patient with a fixed seed. We retained all 8,218 non-epithelial nuclei from the same tumour H5AD as the internal reference, yielding 21,756 epithelial and 8,218 reference nuclei. Ensembl GRCh38 gene positions were obtained from the official BioMart service; 32,364 of 33,538 genes were positioned. We ran infercnvpy 0.6.1 on log-normalised counts with a 100-gene smoothing window, step size 20 and dynamic threshold 1.5. The per-cell CNV score was the mean absolute inferred CNV amplitude. We defined CNV-high using the 99th percentile of the non-epithelial reference scores. This conservative empirical cutoff was anchored to the within-object reference distribution and was not treated as a biological malignancy boundary. Target detection was summarised across patients only when at least 20 CNV-high epithelial nuclei were available. Sensitivity analyses repeated the classification at the 97.5th and 99.5th reference percentiles, included patients with 1–19 CNV-high nuclei, and included all sampled epithelial nuclei irrespective of CNV class. CNV-high is therefore a conservative malignant-cell-compatible indicator, not histological proof of malignancy. The separately deposited normal-bladder object was not integrated or batch corrected with the tumour object; cross-object contrasts were descriptive only. Transcript detection did not establish surface protein, epitope availability, CAR activation or safety. Screening-flow reporting was informed by PRISMA 2020, although this was not an intervention-effect review (17).

### Antigen-pair coverage

For each cohort and scale-specific expression threshold, we calculated single-target prevalence, union coverage for an OR-gated strategy and joint-positive coverage relevant to AND-gated strategies. Because low absolute thresholds saturated coverage, the sensitivity analysis used gene-specific within-cohort 50th, 75th and 90th percentiles. This relative-threshold design compares complementarity but does not define a biological positivity threshold. Pair ordering was evaluated across thresholds and cohorts. These analyses estimate transcript-defined theoretical coverage and do not establish surface-protein abundance, epitope accessibility or CAR function.

### Evidence-gate synthesis

We replaced the original aggregate ordinal scores with two sequential evidence gates. Antigen eligibility required four elements: epitope-matched surface expression on malignant cells at a density linked to CAR activation; patient-level coverage and heterogeneity assessment; assessment of the same epitope in normal urothelium and safety-critical organs; and functional killing assays using relevant normal cells. Only candidates meeting this minimum target definition advanced to product-route validation. This second gate required route-specific tumour exposure, retention or persistence, systemic escape, repeat-dose feasibility, efficacy in a model matching the intended disease distribution, and appropriate local and systemic safety readouts. The gate order is procedural. Route can mitigate local or systemic exposure, but it cannot establish tumour epitope expression or convert missing normal-tissue evidence into demonstrated safety. Failure to satisfy the first gate denotes insufficient evidence, not demonstrated toxicity. Public RNA, Human Protein Atlas records, non-comparative animal models and registry status could identify evidence gaps but could not independently satisfy either gate.

We therefore reported domain-level observations and a route-evidence matrix without weights, means or league-table ranks. A local route was not assumed to improve CAR recognition or efficacy; it was credited only when exposure or outcome was measured directly for the same product and model.

### Statistical analysis and reproducibility

Analyses used version-controlled scripts. We generated descriptive estimates within each cohort and did not use cell-level P values to imply patient-level certainty. For TCGA tumour-versus-adjacent-normal comparisons, we reported median differences, Cliff’s delta and percentile bootstrap 95% confidence intervals from 5,000 resamples with a fixed seed. CNV sensitivity analyses repeated the classification at the 97.5th and 99.5th percentiles of the reference scores. We compared target ordering with the primary 99th-percentile analysis using Spearman rank correlation. We assessed the patient inclusion rule by including any patient with at least one CNV-high nucleus and, separately, all sampled epithelial nuclei. For the normal object, we summarised library size and detected-gene counts by deposited batch and then standardised target detection by equal weighting across pooled library-size quartiles. Because the two bulk cohorts differed in assay processing, platform and disease state, we presented their target orderings descriptively and did not interpret them as evidence of stage robustness. We did not test an aggregate target or platform score. Source identifiers, processing steps, software versions and derived source-data tables accompany the analysis. Synthetic fixtures used for code testing were kept separate from research outputs and did not contribute to any manuscript result.

## Results

### The urothelial-carcinoma CAR evidence base remains predominantly preclinical

The four locked PubMed searches yielded 57 unique records. Dual independent title-and-abstract screening identified 10 core primary studies, six records requiring full-text classification, 33 reviews retained only for overlap assessment and citation chaining, and eight excluded records. Full-text review added a pan-solid c-MET/PD-1 CAR-T study because it included functional T24 bladder-cancer assays; the other five records were non-CAR or contextual evidence without a bladder CAR model. PubMed therefore contributed 11 core primary studies. Combined OpenAlex and Crossref discovery yielded 674 unique candidates and identified one additional 2026 peer-reviewed primary study that was not present in the PubMed candidate set, five independent bladder-specific conference records, two conference abstracts linked to the subsequently published MUC16 article, and one hypothesis paper; 659 records were independently screened at title level by both reviewers and six duplicated PubMed records. During reconciliation and supplementary discovery, the reviewers identified one eligible 2025 Nectin4 CAR-T Original Article outside the original discovery output. The omission occurred because broad relevance-ranked discovery exports were limited to their top 100 records and the article was not indexed by the locked PubMed query; it was found during citation-led reconciliation. The 27 August rerun returned one new PubMed review and no new eligible primary study in the dated PubMed, OpenAlex or Crossref comparison. The reconciled core set therefore comprised 13 primary studies: 11 conventional or adaptor CAR T-cell studies, one CD24-directed CAR-NK study and one CAR-like chimeric PD-1 receptor T-cell study. Six included an *in-vivo* bladder-cancer component, two used patient-derived organoids without an *in-vivo* therapeutic study, and five remained at a cell-line or spheroid level (**Table 1**; **Supplementary Table S1**; **Supplementary Data S1**; **Figures 1**, **2**). No peer-reviewed study reported the treatment of a patient with urothelial carcinoma using a CAR-engineered cell product.

**Table 1:** Peer-reviewed CAR-engineered cellular-therapy evidence in urothelial or bladder cancer.

Study	Target/product	Bladder-specific model level	Route evaluated	Principal supported finding	Key boundary
Abrahimi et al., 2026 (2)	MUC16-recognising natural-ligand CAR T (MSLN-28z)	Cell models; three patient-derived organoids; two orthotopic xenograft models; localisation and surrogate toxicity experiments	Single-dose intravesical versus dose-matched intravenous (5 × 10^6^ cells)	Intravesical, unlike intravenous, treatment improved survival and reduced tumour burden in both models; imaging supported bladder localisation and surrogate experiments supported reduced systemic escape	Preclinical organ-confined models; no human outcome or target-specific normal-urothelial safety evidence
Chang et al., 2025 (18)	NECTIN4 CAR T	UC lines; subcutaneous xenografts; paired pre/post-enfortumab-vedotin biopsies	Intravenous in xenografts	Activity tracked antigen abundance; PPARγ modulation increased NECTIN4 and CAR activity	Immunodeficient subcutaneous models; systemic safety unresolved
Niu et al., 2025 (19)	Second-generation VHH-NECTIN4 CAR T	SW780 and RT4 bladder lines; U87-MG antigen-negative tumour control	*In vitro*	Greater lysis and cytokine release in NECTIN4-positive bladder lines; no significant effect in U87-MG	No organoid, *in-vivo*, normal-urothelial or human evidence; U87-MG is not a safety control
Variant-histology TM4SF1 study, 2025 (20)	TM4SF1 CAR T	Six bladder lines; UMUC3 and 253JBV xenografts	Systemic in xenografts	TM4SF1 CAR T cells killed positive bladder lines and produced durable responses in selected models	Normal-tissue TM4SF1 liability; no human CAR exposure
Oncofetal chondroitin-sulphate study, 2024 (21)	Transient SpyCatcher CAR T armed with VAR2CSA	UM-UC3 cell line and subcutaneous xenograft	Five intravenous doses	Armed, but not unarmed, cells reduced xenograft growth	Repeated arming required; glyco-epitope cannot be inferred from gene expression
SIA-CIgG study, 2024 (22)	SIA-CIgG CAR T	Bladder lines, antigen-challenge assay and xenograft	Systemic in mice	Antigen-dependent killing and persistence; vorinostat augmented activity	Tumour-associated glycoform, no independent replication or human evidence
B7-H3 organoid study, 2023 (23)	Third-generation CD276 CAR T	T24 and three positive patient-derived organoids; one negative control	*In vitro*	Antigen-positive organoids were lysed and released IFN-γ/IL-2	Small organoid series; no *in-vivo* route or safety evidence
CD24 NK-CAR study, 2023 (24)	Third-generation CD24 CAR-NK	Urological panel including luminal bladder lines	*In vitro*	Reduced viability and induced apoptosis in CD24-positive models	Multi-tumour study; no bladder *in-vivo* or route evidence
MUC1 organoid study, 2021 (25)	HMFG2-CD28-CD3ζ CAR T	Matched cystectomy tissue and three patient-derived organoids	*In vitro*	Migration to and lysis of two MUC1-positive organoids, not negative control	Three patients; binder epitope/glycoform is not represented by total MUC1 RNA
EGFR/CD44v6 study, 2021 (26)	EGFR and CD44v6 CAR T	Four UC lines, benign HBLAK cells and spheroids	*In vitro*	Decitabine increased susceptibility in UC models	Benign HBLAK killing at high E:T ratios; no *in-vivo* therapeutic window
Chimeric PD-1 study, 2020 (27)	PD-1–Dap10–CD3ζ chimeric-receptor T cells	Syngeneic multi-tumour models including bladder cancer	Systemic adoptive transfer	Reduced tumour burden in several murine models	CAR-like ligand receptor; bladder was one component of a pan-tumour study
c-MET/PD-1 study, 2025 (28)	c-MET CAR and PD-1-secreting c-MET CAR T	T24 as one bladder line in a pan-solid study	*In vitro*	PD-1-secreting construct improved proliferation and lysis at E:T 20:1	One bladder line; no bladder *in-vivo* model; inconsistent product terminology
Rapid B7-H3 manufacturing study, 2026 (29)	Rapidly prepared nonactivated B7-H3 CAR T	T24 cell line	*In vitro*	Nonviral transfection of nonactivated T cells produced greater T24 lysis than Free-CAR control	No antigen-negative, blocking or normal-cell control; no *in-vivo* or human evidence

This table includes peer-reviewed primary studies only. Conference abstracts are contextual evidence and are not counted as full articles. “Systemic” describes the reported animal route, not clinical feasibility. Detailed extraction fields and evidence boundaries are provided in **Supplementary Data S1**.

**Figure 1:**
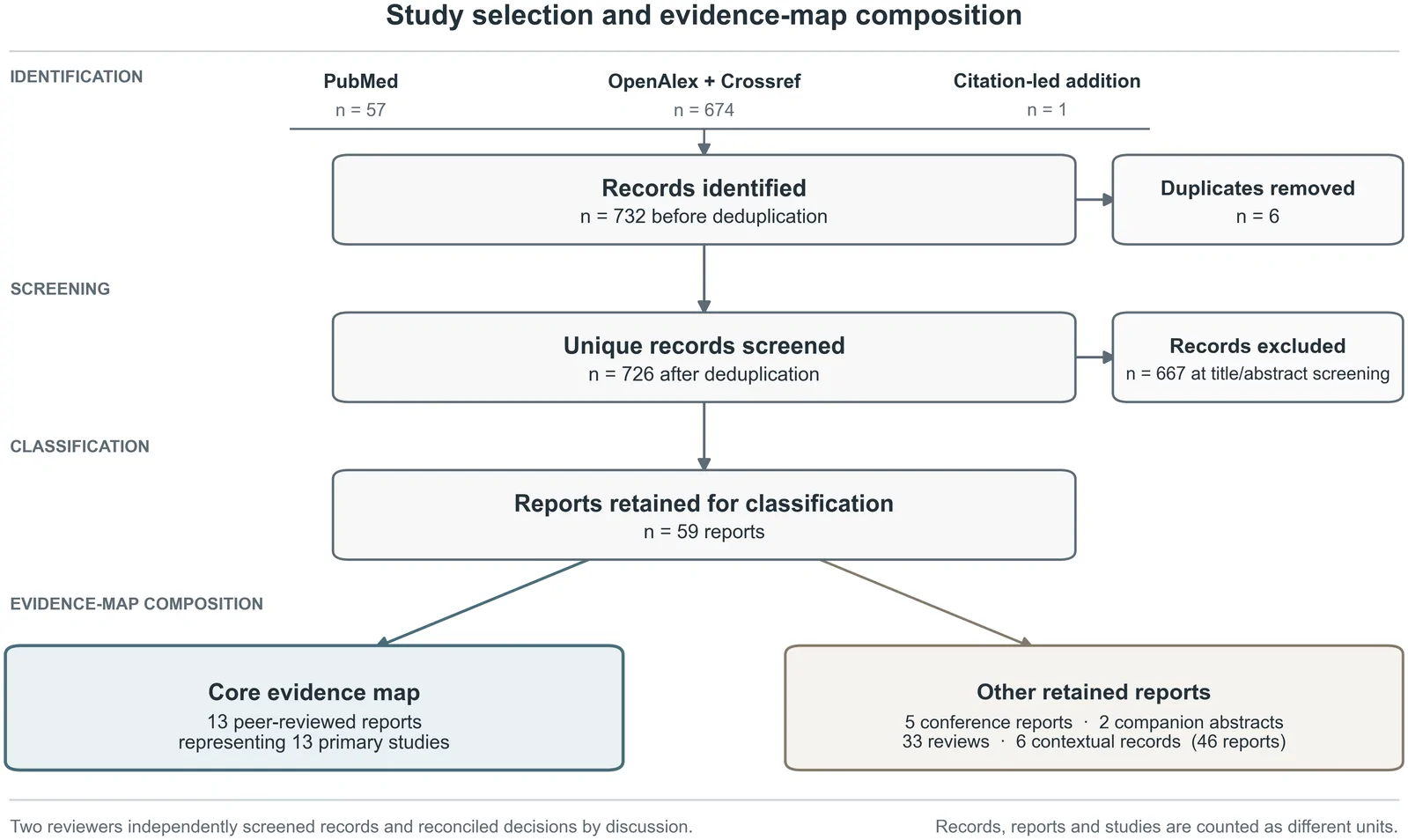
Evidence-flow reconciliation for the urothelial-carcinoma CAR-cell landscape. The evidence map identified 732 records before removal of six cross-source duplicates; 726 records were screened and 667 excluded. Fifty-nine reports were retained for classification: 13 peer-reviewed reports representing 13 core studies, five independent conference reports, two companion reports, 33 reviews and six contextual records. Jiayi Liu and Zhibiao Guo independently screened and reconciled records; one eligible Nectin4 article was identified during citation-led reconciliation outside the relevance-limited discovery exports. Record, report and study counts are separated to prevent double counting and do not imply clinical evidence.

**Figure 2:**
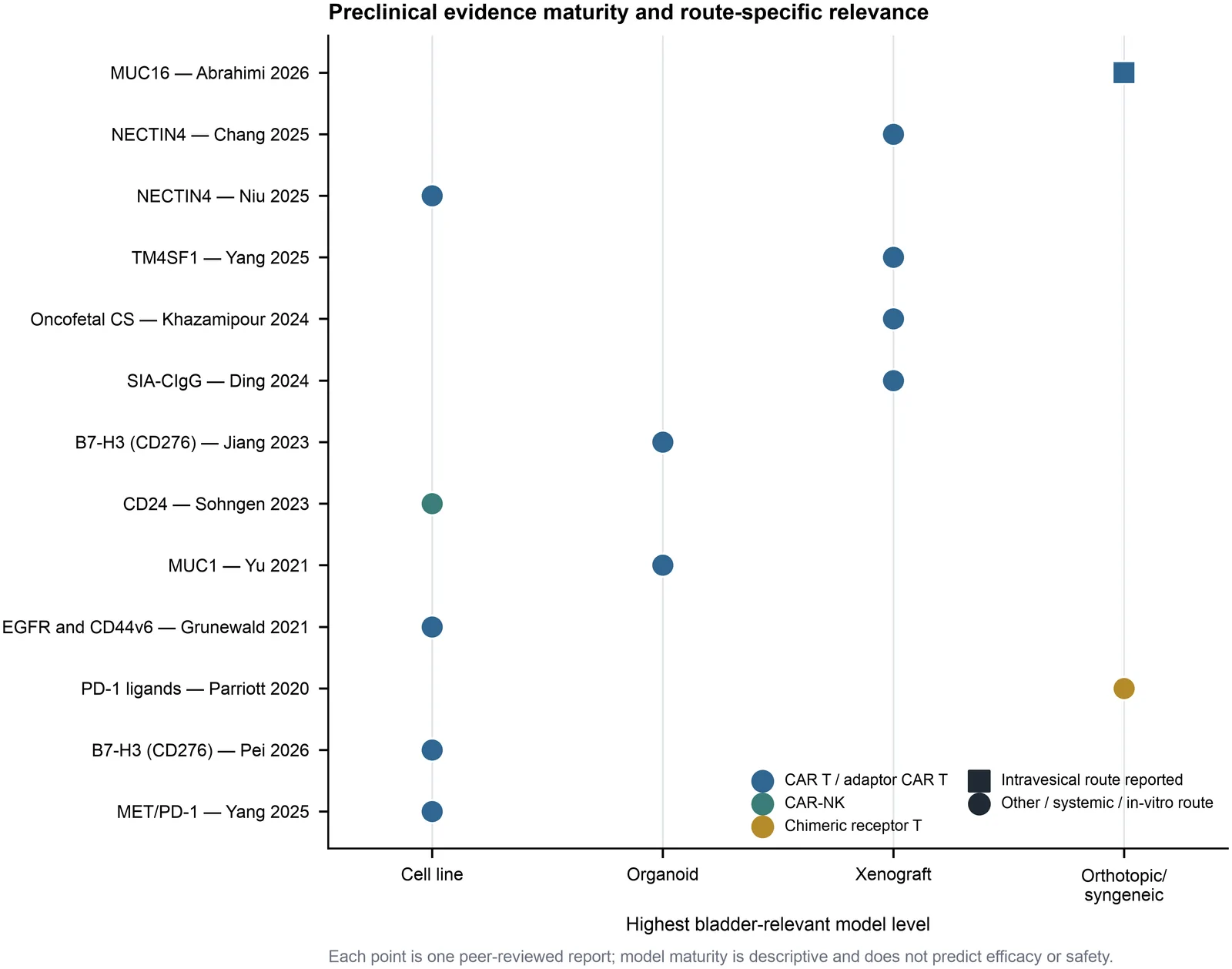
Preclinical evidence maturity and route-specific relevance. Each point represents one peer-reviewed primary report. The horizontal position represents the highest bladder-relevant model level reported: cell line, organoid, xenograft, or orthotopic/syngeneic model. Colour denotes effector platform; square symbols indicate intravesical delivery reported in the study. This descriptive maturity ordering does not estimate comparative efficacy, safety or clinical readiness.

The only direct route comparison was the MUC16-recognising, mesothelin-based CAR T-cell study, for which the publisher full text and supplementary sequences were obtained during revision (2). A single dose of 5 × 10^6^ cells was given intravesically or intravenously in two orthotopic human xenograft models: HT-1376 (n=10 mice per group, pooled across three donors) and UM-UC-3-MUC16TR6 (n=8 per group, two donors). Intravesical treatment improved survival and reduced endpoint bladder/tumour mass in both models, whereas the dose-matched intravenous product did not control outgrowth. Longitudinal imaging showed immediate bladder localisation after intravesical dosing, an estimated residence half-life of approximately 0.8 days and no detectable extra-vesical signal through day 21; peripheral human CD45-positive cells and urinary cytokines were also measured. Surrogate TRP-1 depigmentation and murine CD19 B-cell-aplasia experiments supported reduced systemic escape. These findings are product- and model-specific: they do not establish human safety, MUC16-specific normal-urothelial safety, efficacy in metastatic disease or a general advantage of intravesical administration.

Other studies did not permit a route comparison. A NECTIN4 CAR-T study provided systemic xenograft and target-retention evidence but did not resolve the human therapeutic window (18). A second-generation VHH-NECTIN4 CAR-T study showed greater cytotoxicity and cytokine release in SW780 and RT4 cells than in an antigen-negative U87-MG control but contained no normal-urothelial, organoid or *in-vivo* experiment (19). Systemic xenograft evidence for TM4SF1, oncofetal chondroitin-sulphate and sialylated cancer-derived IgG came from non-clinical models (20–22). B7-H3 and MUC1 studies incorporated small patient-derived organoid sets, whereas EGFR/CD44v6 and CD24 studies did not provide an *in-vivo* bladder-specific safety assessment (23–27). A pan-solid c-MET study used T24 as its only bladder line and provided no bladder *in-vivo* model (28). A 2026 B7-H3 manufacturing study showed greater T24 lysis than a Free-CAR control but included no antigen-negative target, blocking experiment, normal-cell control or *in-vivo* model (29). Conference reports and linked MUC16 abstracts remained contextual and were not counted as peer-reviewed studies (3–5, 30–33).

### Registered programmes address different disease compartments and product strategies

Nine ClinicalTrials.gov records were retrieved. After intervention and integrity review, five were retained: one urothelial-carcinoma-specific CAR T-cell study and four pan-solid-tumour studies that allowed bladder-cancer enrolment. On 24 August 2026, the urothelial-specific phase 1/2 record NCT03185468 changed from recruiting to terminated, with no reason or results posted; its estimated enrolment remained at 20. The four pan-solid programmes evaluated NECTIN4/FAP CAR T cells, HER2 CAR T cells combined with an oncolytic adenovirus, ROR2 CAR T cells and HER2 CAR macrophages. As of 27 August, one was terminated after nine participants across all eligible tumour types, two had unknown registry status and one was recruiting; none provided a bladder-specific participant count or outcome.

Three records were excluded because their interventions were not CAR-engineered cells. A further nominally recruiting NECTIN4/HER2 CAR-NK record, NCT07492628, was excluded from programme counts because its own registry summary described the study as hypothetical and as an example. Registry status was therefore treated as programme metadata rather than evidence of product administration, biological activity or clinical efficacy.

### Abundant candidate transcripts are not equivalent to eligible CAR targets

All 13 prespecified gene-level targets were detected after mapping the former PVRL4 symbol to NECTIN4. ERBB2, PSCA, CD44 and NECTIN4 were among the five most abundant targets in both 407 TCGA muscle-invasive tumours and 89 metastatic GSE176307 cases, whereas MUC16, MSLN and FOLR1 were near the lower end in both cohorts. Because normalisation, platform and disease state differed simultaneously, this descriptive ordering was not interpreted as evidence of stage robustness, equal abundance or therapeutic coverage.

High tumour expression did not imply a favourable bladder tumour-to-normal contrast. In TCGA, median expression was lower in tumour than in adjacent-normal bladder for FOLR2 (median difference, −2.14; bootstrap 95% CI, −2.80 to −1.04), ROR2 (−2.11; −3.42 to −0.94), EGFR (−0.69; −1.03 to −0.13) and CD44 (−0.67; −1.00 to −0.37). By contrast, MUC16 showed the largest positive median difference (1.73; 0.62 to 2.97), followed by MSLN (1.29; −0.25 to 2.62), NECTIN4 (0.76; 0.11 to 3.87), MUC1 (0.74; −0.18 to 2.55), CD276 (0.68; 0.47 to 0.88) and ERBB2 (0.63; 0.02 to 1.75). These comparisons use adjacent bladder rather than systemic normal tissues and therefore cannot establish an on-target/off-tumour safety window. In addition, gene-level MUC1 and CD44 values do not resolve tumour-associated glycoforms or CD44v6.

Current Human Protein Atlas normal-tissue RNA and protein records did not support classifying any gene-level candidate as completely tumour restricted (14). NECTIN4 was group enriched but detected in many tissues; its single-cell reference included urothelial cells, and protein evidence included skin and keratinocytes. ERBB2 and CD44 RNA and EGFR protein were broadly detected; CD276 and MET also had wide normal-tissue profiles. MUC1, MUC16 and MSLN showed glandular, respiratory or mesothelial expression, with secreted or shed-target considerations. FOLR1 showed marked choroid-plexus RNA enrichment together with fallopian-tube, kidney and lung protein evidence; FOLR2 was enriched in placental macrophages; and PSCA showed strong stomach enrichment. These records identify liabilities but do not predict toxicity. For systemic development, the minimum missing evidence is epitope-matched surface quantification across vital organs followed by functional killing with the intended binder. Intravesical development additionally requires normal human urothelial and barrier-injury assays; local delivery cannot be assumed to spare normal mucosa.

### CNV-high epithelial analysis does not identify an unqualified target

The unannotated GSE135337 matrices provided a first sensitivity check rather than cell-type evidence. Across seven bladder-tumour samples, the median fraction of deposited cells with at least one raw count was 0.986 for PSCA, 0.459 for CD44, 0.457 for NECTIN4, 0.293 for ERBB2 and 0.219 for MUC1. MUC16 was absent from the filtered deposited feature matrix. Because cell composition, depth and filtering differed among samples, these values were not interpreted as malignant-cell prevalence, and feature absence was not interpreted as biological absence.

Patient-aware analysis of source-labelled epithelial nuclei showed substantial heterogeneity across 25 patients. We then analysed 29,974 tumour-object nuclei with infercnvpy. The 99th-percentile reference threshold classified 1,828 of 21,756 sampled epithelial nuclei as CNV-high; the proportion varied from 0% to 42.6% by patient. Eighteen patients contributed at least 20 CNV-high epithelial nuclei to target summaries. Median patient-level detection fractions in that subset were 0.398 for ROR2, 0.385 for PSCA, 0.267 for EGFR, 0.266 for MET, 0.229 for CD44, 0.169 for ERBB2, 0.093 for MUC16, 0.083 for NECTIN4 and 0.056 for CD276. MUC1, MSLN, FOLR1 and FOLR2 were each detected in fewer than 0.4% of CNV-high epithelial nuclei overall. At the 97.5th and 99.5th reference percentiles, 22 and 16 patients, respectively, met the 20-nucleus rule. Target ordering remained concordant with the primary analysis at both thresholds (Spearman rho = 0.983; maximum absolute median changes, 0.117 and 0.149, respectively). Six of the seven excluded patients had 1–19 CNV-high nuclei and one had none. Including the six low-count patients yielded 24 evaluable patients and preserved target ordering (rho = 0.989; maximum absolute median change, 0.109). Including all sampled epithelial nuclei from all 25 patients reduced concordance after removal of the CNV restriction (rho = 0.869). This broader analysis did not alter the conclusion that RNA detection alone was insufficient to establish target eligibility. These findings support malignant-cell-compatible localisation for a subset of transcripts but do not constitute histological malignant-cell labels or surface-density measurements.

According to the GSE169379 study design, the four separately deposited normal-bladder batches represented three histologically normal patients. The processed H5AD did not contain a donor-to-batch mapping, so we did not treat the batches as four independent donors. Median urothelial library sizes differed across batches A–D (3,693, 1,974, 3,665 and 2,762 counts per nucleus). Equal weighting across pooled library-size quartiles reduced, but did not eliminate, across-batch detection ranges for EGFR (0.353 to 0.243), PSCA (0.417 to 0.348), CD44 (0.448 to 0.283), ERBB2 (0.307 to 0.116), MET (0.318 to 0.240), NECTIN4 (0.401 to 0.183) and ROR2 (0.299 to 0.177). This attenuation indicated that sequencing depth contributed to batch variation. Raw median urothelial detection fractions across batches were 0.598 for EGFR, 0.527 for PSCA, 0.423 for CD44, 0.401 for ERBB2, 0.324 for MET, 0.290 for NECTIN4 and 0.215 for ROR2. MUC16 and CD276 were less frequently detected in normal urothelium (0.008 and 0.038) than in CNV-high epithelial nuclei (median across eligible patients, 0.093 and 0.056). Neither contrast established a therapeutic window. Donor and processing effects could not be separated, the tumour and normal objects were unpaired, RNA did not measure the CAR epitope, and both targets had extra-vesical liabilities. Across the integrated evidence, no target satisfied the antigen-eligibility gate (**Figure 3**).

**Figure 3:**
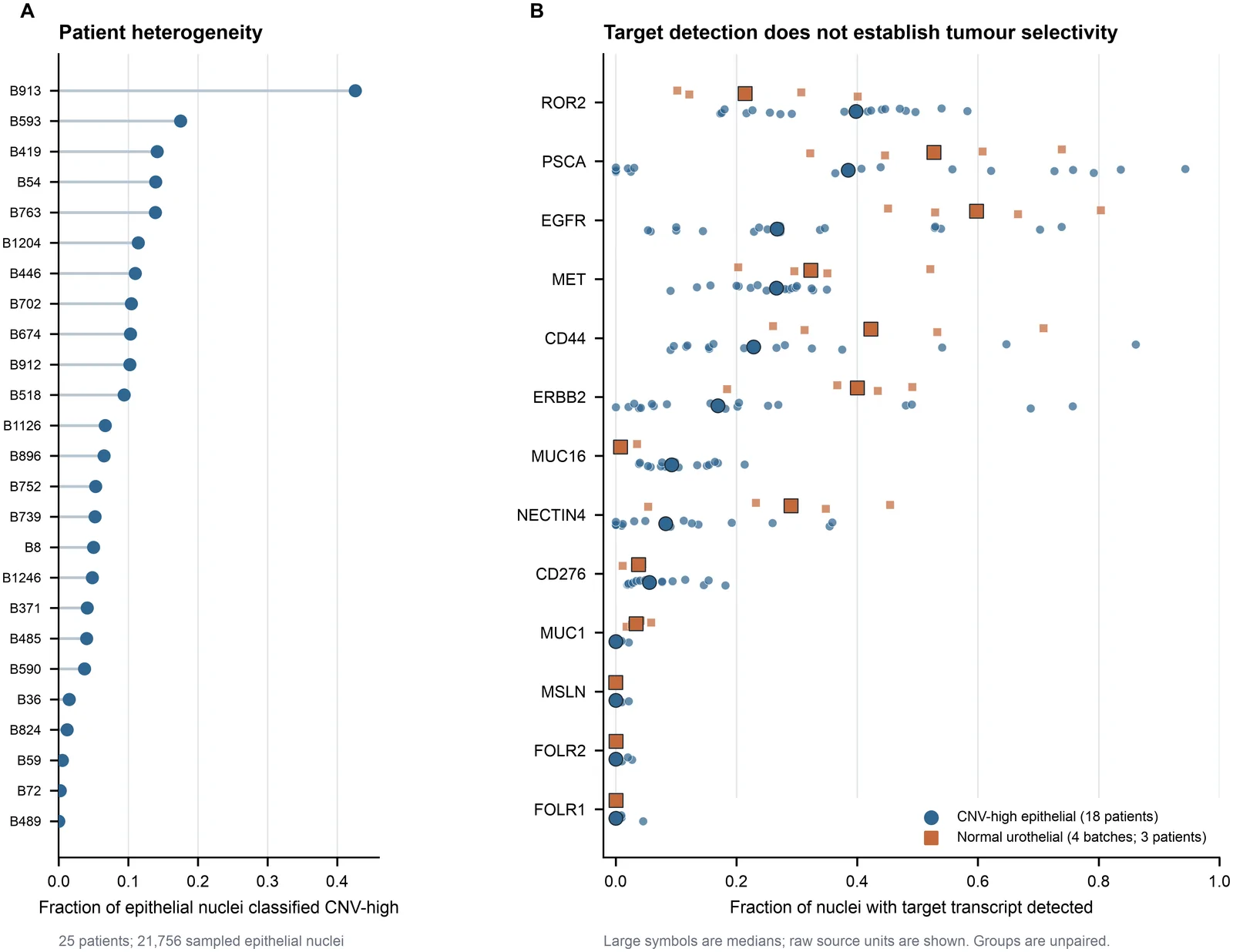
CNV-high epithelial inference and candidate-target RNA detection. **(A)** Fraction of sampled epithelial nuclei classified CNV-high in each of 25 GSE169379 MIBC patients. infercnvpy used all 8,218 non-epithelial nuclei from the same tumour object as the reference; the primary CNV-high threshold was the 99th percentile of reference-cell mean absolute CNV scores. Up to 1,000 epithelial nuclei were sampled per patient with a fixed seed. Threshold and patient-inclusion sensitivity analyses are reported in the Results section and **Supplementary Data S8**; normal-batch depth-standardisation results are provided in **Supplementary Data S9**. **(B)** Target transcript-detection fractions in CNV-high epithelial nuclei from 18 patients with at least 20 such nuclei (blue circles) and in normal urothelial nuclei from four separately deposited batches derived from three patients (orange squares). Small symbols show source-unit values and large outlined symbols show medians. The groups are unpaired and were not integrated or batch corrected. CNV-high is a malignant-cell-compatible indicator, not histological proof, and RNA detection does not establish surface epitope density, CAR activation or safety.

### Transcript-defined antigen pairs remain a sensitivity analysis

Low absolute expression thresholds produced near-universal OR-gated coverage for many pairs. At gene-specific relative quantiles, many pairs tied and the apparent leaders changed with the threshold. These results were therefore retained only as a reproducible sensitivity analysis and were not used to nominate a construct.

Gene-level RNA cannot resolve CD44v6 or tumour-associated glycoforms, define receptor density, show that two antigens coexist on the same malignant cell, or predict gate activation and normal-cell killing. The pair analysis therefore did not satisfy the antigen-eligibility gate and contributes no clinical decision rule.

### Route is a product-specific exposure question after antigen eligibility

Removal of aggregate scores changed the output from a target hierarchy to an evidence-gaps matrix. NECTIN4 had the broadest combination of bladder-specific preclinical evidence and registry relevance, but normal-urothelial detection and absent epitope-matched functional safety prevented prioritisation. MUC16 had the only direct route comparison and a higher descriptive CNV-high-to-normal-urothelial RNA contrast, but low prevalence, shedding-related uncertainty and absent human normal-urothelial testing prevented prioritisation. The other candidates similarly lacked at least one required antigen-eligibility measurement. Thus, no target passed the first gate.

The route-evidence matrix contains no numerical fit score. For a product intended only for organ-confined luminal disease, intravesical development requires measured bladder exposure, mucosal penetration, retention, repeat-dose feasibility, normal-urothelial injury and systemic escape. For disseminated disease, a systemic product requires measured trafficking to relevant metastatic sites, persistence, systemic normal-tissue exposure and activity under metastatic microenvironmental constraints. The MUC16 study supports the feasibility of the first experimental pathway for one construct; no evidence supports generalising that result to other antigens, CAR-NK products, invasive extravesical disease or patients (**Figure 4**).

**Figure 4:**
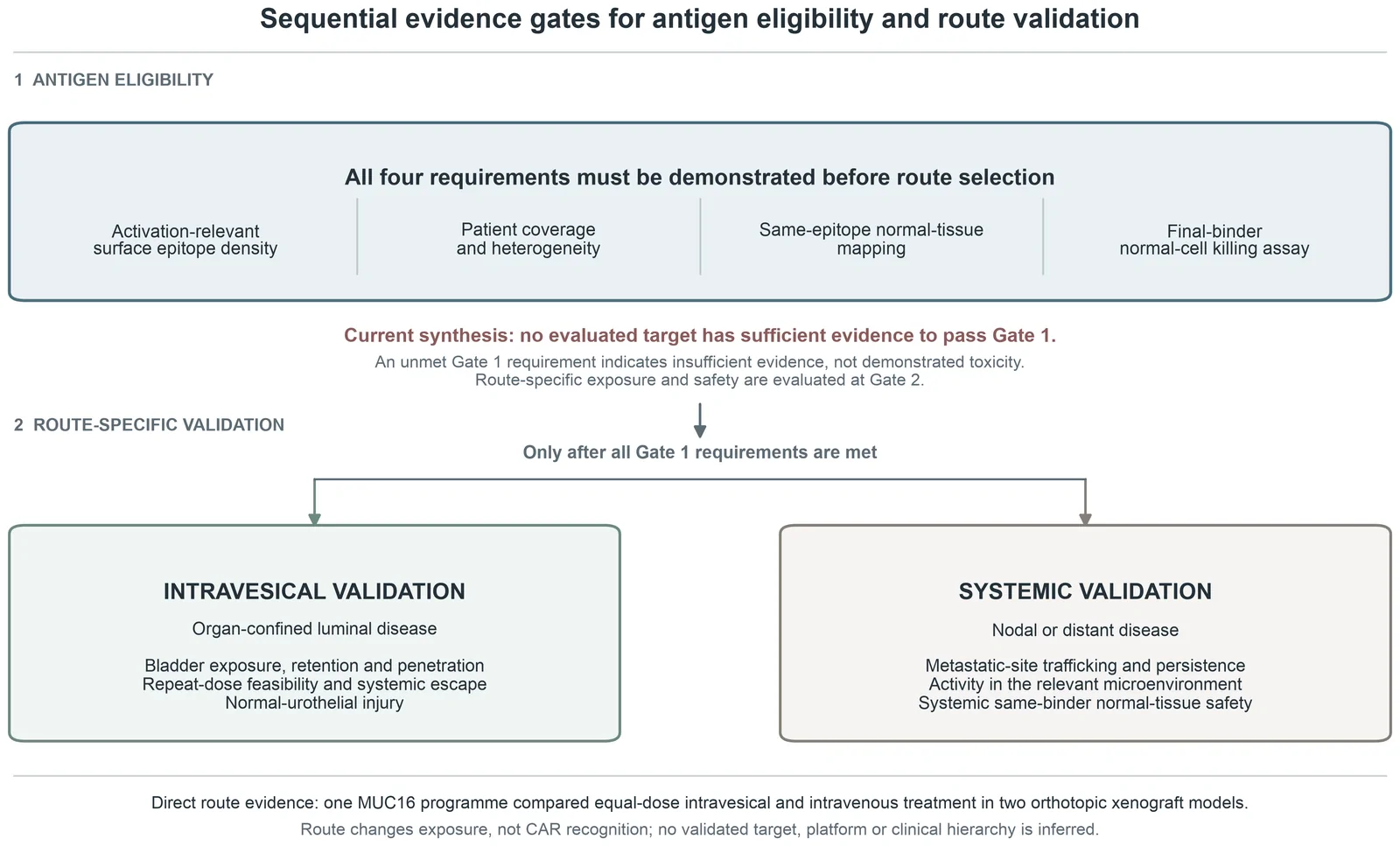
Sequential evidence gates for antigen eligibility and route validation. The first gate requires activation-relevant malignant-cell surface epitope density, patient coverage and heterogeneity assessment, same-epitope normal-tissue mapping and functional normal-cell killing by the final binder. Products that do not satisfy these requirements stop before route selection. After antigen eligibility, intravesical and systemic development require different measured exposure and safety packages for their intended disease distributions. The only direct route evidence was an equal-dose intravesical-versus-intravenous comparison for one MUC16-directed CAR T-cell programme in two orthotopic xenograft models. Route changes exposure, not CAR recognition; the figure contains no target, route or platform score.

## Discussion

### Antigen eligibility precedes route selection

The revised analysis gives a direct conclusion: none of the evaluated targets can yet be recommended or excluded for clinical use, because none has the complete evidence needed to cross the antigen-eligibility gate. CNV-high epithelial analysis improved malignant-cell-compatible localisation, but it did not measure the CAR-recognised surface epitope or demonstrate killing selectivity. The finding that several abundant transcripts were at least as frequent in separately processed normal urothelium makes a quantitative league table less defensible, not more.

The required next evidence is target specific. NECTIN4 programmes need epitope-matched surface quantification and functional killing in normal urothelium and keratinocytes, together with post-enfortumab assessment where relevant. ERBB2 and EGFR require the same-binder evaluation across vital epithelial tissues; CD44 programmes require isoform-specific CD44v6 measurements rather than total CD44; PSCA programmes require stomach and urothelial safety assessment. MUC16/MSLN constructs require measurement of membrane epitope density, soluble-antigen interference and mesothelial, respiratory and normal-urothelial killing. These tests must use the final CAR binder and an activation-relevant density range. Public RNA or generic immunohistochemistry cannot substitute for them.

Patient-derived bladder organoids offer a model for studying interpatient heterogeneity and treatment response. Bladder-organoid biobanks can retain parental histological and genomic features and support drug-response profiling. However, culture-associated clonal evolution and incomplete correspondence with *in vivo* response remain important limitations (36, 37). For CAR development, tumour organoids could assess epitope retention, heterogeneity, target-dependent killing and antigen escape. Matched normal urothelial organoids could provide a complementary assessment of on-target, off-tumour effects before orthotopic *in vivo* confirmation. Findings from these models would not by themselves establish CAR-cell efficacy or safety.

### Route changes exposure, not CAR recognition

The full MUC16 report materially strengthens one narrow inference. At an equal cell dose, intravesical administration produced bladder-localised exposure and better tumour control than intravenous administration in two orthotopic xenograft models. This is direct evidence that route altered biodistribution and outcome for that product in those models. It does not show that compartment determines CAR efficacy, that another construct will behave similarly, or that normal human urothelium is protected.

Accordingly, route is the second gate. An intravesical programme for organ-confined luminal disease should quantify viability in urine, tumour-surface distribution, mucosal penetration, dwell time, repeat-dose performance, normal-urothelial and barrier injury, and escape into blood and extra-vesical organs. A systemic programme for nodal or distant disease should quantify trafficking to relevant metastatic sites, expansion and persistence, activity in suppressive microenvironments and exposure of every safety-critical normal tissue carrying the epitope. CAR T, CAR-NK and myeloid platforms may differ in manufacturing and persistence, but the present urothelial evidence does not establish a platform hierarchy.

### The framework is a stopping rule, not a ranking

The framework yields a two-stage stopping rule. The order is procedural rather than mechanistic. Delivery route can alter local retention, systemic escape and normal-tissue exposure, but route optimisation requires a defined product. The recognised epitope, tumour coverage and normal-tissue distribution of that product must first be characterised. If malignant-cell surface density and same-binder normal-tissue safety remain undemonstrated, development should pause because the evidence is insufficient, not because toxicity has been shown. We reserve the term demonstrated toxicity for direct functional or *in vivo* observations. After antigen eligibility is established, the proposed route must show product-specific exposure and efficacy in a model matching the intended disease distribution. This framework explains why NECTIN4 can have the broadest evidence coverage without being declared ready and why MUC16 can have the strongest route experiment without becoming the preferred target.

### Limitations

This analysis has several limitations. First, no peer-reviewed human urothelial-carcinoma outcome was identified. Although two reviewers independently screened and reconciled records, one eligible Nectin4 article was found by citation-led reconciliation outside the relevance-limited discovery exports. The dated 27 August rerun found no additional eligible primary study, but retrieval incompleteness remains possible in a rapidly moving field. Conference abstracts may change after full publication, and registry status does not prove dosing or activity (34, 35).

Second, molecular cohorts differed in disease state, assay and sampling. TCGA adjacent bladder is neither healthy urothelium nor a systemic safety panel. GSE176307 used a different expression scale, so we did not interpret concordant target ordering as evidence of stage robustness. The GSE169379 CNV-high label was based on inferred broad chromosomal expression patterns and internal non-epithelial references; it was not validated against pathology, mutation or genotype. Alternative thresholds and patient-inclusion rules preserved target ordering but changed some median fractions, showing that the numerical estimates were sensitivity-dependent. The four normal batches came from three patients, donor-to-batch mapping was unavailable, and depth standardisation attenuated but did not remove batch heterogeneity. The tumour and normal objects were unpaired and separately processed. Dropout, ambient RNA, depth and other processing effects remain relevant. Neither RNA nor Human Protein Atlas records can establish epitope-matched surface density or functional toxicity.

Third, antigen-pair coverage used relative within-gene quantiles and remains a transcript-complementarity sensitivity analysis, not a biological CAR threshold. We removed aggregate target and platform-route scores, but the replacement evidence gates are still development criteria rather than clinically validated biomarkers. They require prospective experimental testing and cannot be used for patient selection.

## Conclusions

The urothelial-carcinoma CAR evidence base remains preclinical. No candidate presently meets the requirements for unqualified target prioritisation. Development should first demonstrate activation-relevant malignant-cell surface expression and same-binder functional safety in normal tissues. Only then should a local or systemic route be selected and tested for the exposure required by the intended disease distribution. The MUC16 study supplies product-specific intravesical proof of principle; it does not establish a general route or platform hierarchy. Epitope-matched validation, functional normal-tissue testing, route-specific biodistribution and auditable human outcomes are the decisive next evidence.

## Data Availability

All derived data, source-data tables, analysis scripts, scoring-removal audit materials and provenance records that can legally be redistributed are available in the versioned Zenodo repository at https://doi.org/10.5281/zenodo.21422757. The public source datasets remain available from TCGA/UCSC Xena, GEO (GSE176307, GSE135337 and GSE169379) and the Human Protein Atlas under their source terms. Third-party publisher full texts are not redistributed.
