# Supplementary material for: Evidence gaps and route-dependent development requirements for CAR cellular therapy in urothelial carcinoma: a systematic evidence map and public-data analysis
Source: Front Immunol. 2026 Oct 22;17:1941701. doi: 10.3389/fimmu.2026.1941701 (PMC13735611; doi:10.3389/fimmu.2026.1941701)
Supplement: Supplementary file 1 [file Table2.docx]

Supplementary Information

**Evidence gaps and route-dependent development requirements for CAR cellular therapy in urothelial carcinoma**

*Revised Supplementary Information for Frontiers in Immunology Original Research*

# Review design

We conducted a systematic evidence map of CAR-engineered immune-cell approaches in bladder cancer and urothelial carcinoma. The dated analytic protocol was locked on 15 July 2026 before quantitative results were inspected. The review was designed to identify development-relevant evidence, rather than to pool therapeutic efficacy: eligible evidence includes preclinical CAR-cell studies, registered interventional trials and public molecular datasets; no meta-analysis of intervention effects was planned.

# Information sources and last search dates

- MEDLINE/PubMed: four searches from database inception through 17 July 2026; all four were rerun through the official API on 27 August 2026.
- Crossref and OpenAlex: four supplementary discovery queries each, completed on 17 July 2026 and rerun on 27 August 2026.
- ClinicalTrials.gov: public API retrieval on 17 July 2026 and status/integrity update on 27 August 2026.
- Reference and forward-citation screening: included primary studies and recent overlapping reviews.

The exact executable query strings, raw machine-readable exports, timestamps and retrieval logs are preserved in the associated reproducibility archive and its provenance manifests.

The locked screening set remained the basis of the flow diagram. The revision rerun returned 58 unique PubMed records, including one newly indexed review but no new eligible primary study. The dated Crossref/OpenAlex comparison likewise found no new eligible urothelial CAR-cell primary study. ClinicalTrials.gov returned the same nine candidate records; NCT03185468 had changed from recruiting to terminated on 24 August 2026, without a posted reason or results.

# Core PubMed query concepts

The primary query combined controlled vocabulary and title/abstract terms for: (1) bladder neoplasms or urothelial carcinoma; and (2) chimeric antigen receptors, CAR T cells, CAR-NK cells, CAR-gamma-delta cells or CAR macrophages. Supplementary PubMed queries targeted locoregional administration, candidate antigens and review overlap. No date, language or publication-type limit was applied during retrieval.

# Eligibility criteria

We included peer-reviewed in-vitro, organoid, in-vivo, translational and clinical studies directly testing CAR-engineered immune cells in bladder cancer or urothelial carcinoma. Registered interventional trials were retained irrespective of recruitment status but were not interpreted as evidence of dosing, activity or efficacy. Conference abstracts were retained separately only if no full report was identified. Reviews supported citation chaining and novelty-overlap assessment but never substituted for primary evidence.

We excluded conventional NK-cell, tumour-infiltrating lymphocyte, T-cell-receptor-engineered and immune-engager studies without a CAR component, as well as non-urothelial CAR studies without a urothelial model or directly applicable target-safety evidence. Upper-tract evidence was recorded separately and was not combined with bladder-specific quantitative analyses unless separable data were available.

# Deduplication, screening and linkage

Records were deduplicated in the following order: DOI, PMID, then normalised title. We linked preprints, conference abstracts, registry records and full publications when they described the same product, target, route, model and author group. A conference report was not counted as an independent study when a linked peer-reviewed article was identified.

Title/abstract exclusions are documented in the reproducibility archive's PubMed and supplementary screening-audit tables. The reconciled post-deduplication denominator is 726 records. Jiayi Liu and Zhibiao Guo independently screened all records at title/abstract and full-text stages against the prespecified eligibility criteria, then reconciled screening decisions by discussion. Both reviewers independently screened the 659 supplementary provisional title exclusions. During reconciliation and supplementary discovery, they identified one eligible Nectin4 CAR-T Original Article outside the original discovery output; its publisher full text was verified and the article was added to the evidence base. The completed dual-reviewer screening and reconciliation record is retained with the final screening materials.

# Data extraction and evidence-maturity boundary

For each included record we extracted disease setting, target, platform, donor source, CAR design, route, model, comparator, sample information, safety observations, antitumour readouts, publication status and human-outcome availability. Evidence maturity was described qualitatively as cell line, organoid/three-dimensional culture, subcutaneous xenograft, orthotopic/syngeneic model, registered human dosing and peer-reviewed human outcome. This hierarchy is a translational-proximity description, not a validated risk-of-bias scale.

# Single-nucleus copy-number inference

The treatment-naive GSE169379 tumour object contained 67,988 nuclei from 25 patients. Up to 1,000 source-labelled epithelial nuclei per patient and all 8,218 same-object non-epithelial nuclei were retained, yielding 21,756 epithelial and 8,218 reference nuclei. Ensembl GRCh38 gene positions were retrieved from the official BioMart service; 32,364 of 33,538 genes were positioned. infercnvpy 0.6.1 was run on log-normalised counts with a 100-gene smoothing window, step size 20 and dynamic threshold 1.5. The per-cell score was the mean absolute inferred CNV amplitude. Epithelial nuclei above the 99th percentile of the non-epithelial reference score distribution (0.0138714429) were labelled CNV-high. A patient contributed to target summaries only when at least 20 CNV-high epithelial nuclei were available.

The analysis classified 1,828 epithelial nuclei as CNV-high; 18 patients met the target-summary threshold. This is a malignant-cell-compatible label, not histological proof. The separately deposited normal-bladder object was processed without integration or batch correction against the tumour object. Therefore, tumour-versus-normal RNA contrasts are unpaired descriptive comparisons and cannot establish surface epitope density or safety. Threshold and patient-inclusion sensitivity results are supplied in Supplementary Data S8. Normal urothelial batch library sizes and library-size-standardised detection ranges are supplied in Supplementary Data S9. The four normal batches derive from three patients; donor-to-batch mapping is unavailable. Standardisation equally weights pooled library-size quartiles and does not establish a therapeutic window.

# Registry-integrity procedure

ClinicalTrials.gov records were assessed for intervention identity, disease eligibility, route, phase, status and posted outcomes. A record that identified itself as a hypothetical/example study despite a recruiting label (NCT07492628) was excluded from programme counts. The anomaly, dated status changes and raw source text are preserved in the associated reproducibility archive.

# Reproducibility and interpretation boundaries

All manuscript-facing tables and figures were regenerated from versioned scripts. Dated search responses, the BioMart gene-position table, inferCNV run metadata, patient-level and target-level summaries, figure source data and SHA-256 manifests are included in the versioned repository. Publisher full texts and other third-party copyrighted files are not redistributed. Aggregate target and platform-route scores used in the initial submission were withdrawn during revision and are not included as revised supplementary results.

# Reporting note

This supplement is aligned with applicable PRISMA 2020 reporting domains, while transparently stating where the present work differs from an intervention-effect systematic review. The completed dual-reviewer screening and reconciliation record, 726-record/13-study locked flow and 27 August 2026 update audit are retained in the submission package.

# Table S1. Auditable screening-flow counts

| **Flow stage** | **Count** | **Accounting note** |
| --- | --- | --- |
| Records unique within the four PubMed searches | 57 | Searches rerun to 17 July 2026; includes primary studies, reviews and records requiring full-text classification |
| Supplementary Crossref/OpenAlex candidates unique within supplementary discovery | 675 | Four Crossref and four OpenAlex searches rerun/completed 17 July 2026, plus one eligible article identified during dual-reviewer reconciliation and supplementary discovery |
| Records before cross-source deduplication | 732 | 57 + 675 |
| Duplicate PubMed records found in supplementary discovery | 6 | Removed from combined screening denominator; linkage retained in the supplementary screening-audit table in the reproducibility archive |
| Records screened after cross-source deduplication | 726 | 732 − 6 |
| Peer-reviewed primary studies included in the core evidence set | 13 | 11 from PubMed after full-text classification, one Current Urology report found in supplementary discovery and one Nectin4 article identified during dual-reviewer reconciliation and supplementary discovery |
| Independent bladder/urothelial conference reports retained separately | 5 | Not counted as peer-reviewed primary studies or as evidence of clinical activity |
| Companion conference reports linked to the subsequent MUC16 full article | 2 | Retained for provenance but not counted as independent studies |
| Context/hypothesis records retained without inclusion in core evidence | 6 | Five contextual/non-CAR records resolved during PubMed full-text review plus one hypothesis record from supplementary discovery |
| Reviews retained for overlap assessment/citation chaining | 33 | Not substitutes for primary evidence |
| Records excluded after screening | 667 | 8 PubMed exclusions + 659 supplementary title exclusions after dual independent human screening and reconciliation |

# Supplementary Table S2. Guide to non-restricted supplementary data

| **File** | **Contents** | **Interpretation boundary** |
| --- | --- | --- |
| Supplementary Data S1 | Record-level extraction for the 13 peer-reviewed primary studies | Descriptive evidence extraction; model maturity and route do not predict clinical efficacy or safety. |
| Supplementary Data S2 | Dated ClinicalTrials.gov status and integrity audit | Registry metadata do not establish dosing, activity or efficacy. NCT07492628 is excluded because its official summary describes a hypothetical/example study. |
| Supplementary Data S3 | Patient-level epithelial and reference sampling plus CNV-high fractions in GSE169379 | CNV-high is a malignant-cell-compatible inference, not pathology-confirmed malignancy. |
| Supplementary Data S4 | Target-transcript detection by patient among CNV-high epithelial nuclei | RNA detection does not establish the CAR-recognised surface epitope, activation threshold or therapeutic coverage. |
| Supplementary Data S5 | Target-transcript detection by batch in separately deposited normal urothelium | Tumour and normal objects are unpaired and separately processed; the contrast cannot establish safety. |
| Supplementary Data S6 | Quantile-based antigen-pair sensitivity analyses in TCGA-BLCA and GSE176307 | Transcript-defined theoretical coverage; not a biological positivity threshold, surface-protein measurement or gate-function test. |
| Supplementary Data S7 | Human Protein Atlas-informed normal-tissue liability screen | RNA/protein atlas evidence does not establish epitope-matched surface density or functional toxicity. |
| Supplementary Data S8 | CNV threshold and patient-inclusion sensitivity: evaluable patient counts, Spearman correlations and maximum absolute median detection-fraction changes | Alternative empirical thresholds and inclusion rules assess sensitivity; they do not validate malignancy or establish surface epitope density. |
| Supplementary Data S9 | Normal urothelial batch library sizes and raw versus library-size-standardised target-detection ranges | Four batches derive from three patients; donor-to-batch mapping is unavailable. Depth adjustment does not remove donor or processing effects or establish safety. |

# PRISMA 2020 working checklist

| **PRISMA domain** | **Location / evidence** | **Status** | **Submission boundary** |
| --- | --- | --- | --- |
| Title identifies review approach | Manuscript title | Complete | Retain “systematic evidence map and public-data analysis” |
| Structured abstract | Manuscript Abstract | Complete | Check target-journal word limit and abstract format |
| Rationale and objectives | Introduction | Complete | Objective explicitly avoids clinical efficacy inference |
| Eligibility criteria | Methods; Supplementary Methods 1 | Complete | Conference and registry evidence remain separately labelled |
| Information sources and search dates | Methods; Supplementary Methods 1; dated search logs and revision update record | Complete as of 27 August 2026 | Record any later delta screen if resubmission is materially delayed |
| Full search strategies | Reproducibility archive search-strategy record | Complete | Export in journal supplement format |
| Selection process | Methods; completed dual-reviewer screening and reconciliation record | Complete | Jiayi Liu and Zhibiao Guo independently screened records at title/abstract and full-text stages. Both independently screened the 659 supplementary provisional title exclusions; decisions were reconciled by discussion. |
| Data collection / extracted items | Methods; core extraction table | Complete | Retain documented full-text evidence boundaries before lock |
| Study-risk/evidence assessment | Methods; Table 1; maturity boundaries | Complete for landscape purpose | This is not a validated risk-of-bias instrument |
| Synthesis methods and sensitivity analyses | Methods; inferCNV outputs; antigen-pair sensitivity table; evidence-gate matrix | Complete | Aggregate target and platform-route scores were withdrawn; preserve descriptive boundaries |
| Selection results and flow diagram | Table S1; Figure 1; source-data tables | Complete | The final reconciliation is 726 records and 13 peer-reviewed core studies. Retain the completed dual-reviewer screening and reconciliation record. |
| Study characteristics | Table 1; core extraction TSV | Complete | Current Urology full-text audit completed 17 July 2026; retain its strict evidence boundary |
| Results of syntheses | Results; source-data tables; four revised figures | Complete | Preserve patient-level summaries and unpaired cross-object boundary |
| Certainty / limitations | Discussion and Limitations | Complete | Do not overstate transcript or preclinical evidence |
| Registration, protocol and support | Methods; dated protocol and deviations; author declarations | Complete | The evidence-map protocol was prospectively time-stamped locally but not registered in a public review registry; funding, competing interests, CRediT and AI-use statements are reported |
| Data, code and materials availability | Data availability statement; versioned Zenodo record | Revision archive prepared; publication of the new Zenodo version remains an author portal action | Cite the stable concept DOI 10.5281/zenodo.21422757, which resolves to the latest published version |

This checklist is a transparency aid for a systematic evidence map, not evidence of an intervention-effect meta-analysis.
